# GeneRank: Using search engine technology for the analysis of microarray experiments

**DOI:** 10.1186/1471-2105-6-233

**Published:** 2005-09-21

**Authors:** Julie L Morrison, Rainer Breitling, Desmond J Higham, David R Gilbert

**Affiliations:** 1 Bioinformatics Research Centre, University of Glasgow, Glasgow, UK; 2 Department of Mathematics, University of Strathclyde, Glasgow, UK; 3 Institute of Biomedical and Life Sciences, Glasgow, UK

## Abstract

**Background:**

Interpretation of simple microarray experiments is usually based on the fold-change of gene expression between a reference and a "treated" sample where the treatment can be of many types from drug exposure to genetic variation. Interpretation of the results usually combines lists of differentially expressed genes with previous knowledge about their biological function. Here we evaluate a method – based on the PageRank algorithm employed by the popular search engine Google – that tries to automate some of this procedure to generate prioritized gene lists by exploiting biological background information.

**Results:**

GeneRank is an intuitive modification of PageRank that maintains many of its mathematical properties. It combines gene expression information with a network structure derived from gene annotations (gene ontologies) or expression profile correlations. Using both simulated and real data we find that the algorithm offers an improved ranking of genes compared to pure expression change rankings.

**Conclusion:**

Our modification of the PageRank algorithm provides an alternative method of evaluating microarray experimental results which combines prior knowledge about the underlying network. GeneRank offers an improvement compared to assessing the importance of a gene based on its experimentally observed fold-change alone and may be used as a basis for further analytical developments.

## Background

Since its launch in 1998, the Google search engine has all but monopolized page searches on the world-wide web [1]. The basis of this astonishing success is the PageRank algorithm developed by Google founders Larry Page and Sergey Brin [2], which allows efficient and stable prioritization of search results. Here we show how the basic idea of PageRank can be transferred quite intuitively to the analysis of gene expression datasets in molecular biology. We modify PageRank appropriately to produce a new algorithm, GeneRank, and explore its limits and potential for the analysis of both synthetic and real-world data.

Just as the original PageRank is stable against the artificial inflation of a web page's rank by web designers, we hope that GeneRank may obtain a more robust ranking of genes in (typically very noisy [3]) microarray experiments. While PageRank uses hyperlinks between web pages to achieve this end, we combine the expression measurements with external information, such as functional annotations, protein interaction data or previous experimental results.

Data sharing techniques have been successfully implemented previously using, for example, GO annotations [4,5] or protein-protein interactions [6-8] to define the network connectivity. Justification for this is given by the observation that connected genes are more likely to be co-expressed [6]. Additional advantages include the possibility for GO terms to provide insight into the process of co-expression [4] and for functional classification to be made for previously unannotated genes [7,9]. These examples serve to demonstrate the value and feasibility of combining data from different sources. Not only is the analysis of a microarray experiment likely to be more robust when prior information is included, but networks rather than single genes can be identified as important, and further biological inferences can be drawn about the data with the aid of this added information.

Our aim in this work is to use connectivity data to produce a prioritization of the genes in a microarray experiment that is less susceptible to variation caused by experimental noise than one based on expression levels alone. This is achieved using GeneRank, a customised version of the PageRank algorithm.

## Results and discussion

### The algorithm

**Figure 1:**
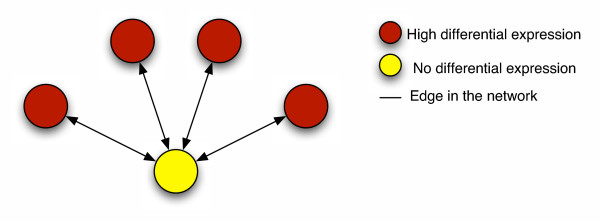
**The Vote of Confidence Principle. **Just as a the PageRank of a web page will be high if it is linked to other highly ranked pages, we hope that the relative ranking of a gene will be increased if it is linked to other highly differentially expressed genes.

The algorithm on which we base our method of microarray experiment analysis was originally devised for assessing the importance of web pages in search engine results. We show here that its formulation allows for a simple and intuitive extension for our application. The PageRank algorithm, used by the successful search engine Google [10], is based on the premise that a web page should be highly ranked if other highly ranked pages contain hyperlinks to it. This idea naturally extends to analysing the results of a microarray experiment, where we would like a gene to be highly ranked if it is linked to other highly ranked genes, even if its own position is lower, e.g., due to measurement variability. We can think of this as the "vote of confidence" principle (See Figure 1). Here we would hope that the relative ranking of the gene with little or no differential expression will be boosted by the PageRanking process. In some cases there may be an even stronger biological interpretation. For example, suppose the gene with low differential expression is a transcription factor that controls the expression of all genes connected to it. The transcription factor itself may be "activated" by the experimental treatment but not change its expression – but its target genes will. Hence, GeneRank should be able to highlight the transcription factor among the results.

The original PageRank algorithm also has a random walk interpretation where the ranks correspond to the invariant measure of a teleporting random walk on the web. This is equivalent to saying that the rank of a web page is proportional to the time spent at the web page whilst surfing the web. This idea can also be intuitively extended to ranking genes, where the rank of a gene is proportional to the amount of time a biologist should spend looking at a gene whilst analysing the experimental results.

As with the original algorithm, we require a network or graph to allow us to calculate a rank for each entity in the network. With the original algorithm, nodes represent web pages and a link exists between two nodes if one page contains a hyperlink to the other. This results in a directed graph. In our case, we define an undirected graph where a node represents a gene and the edges can be defined by some other "previous knowledge". For our purpose, we use either Gene Ontology annotations [11] or expression profile correlation coefficients. In addition to the network structure, we require a vector of expression changes, experimentally observed in a microarray experiment, as input for the algorithm. Other measures of differential expression could also be used, e.g. p-values, which are suitably transformed to ensure genes which are highly changed have a large input value. We shall only consider the use of expression changes.

For each gene, the expression level vector contains the value for its expression change in the experiment under consideration. The algorithm, GeneRank, also uses a free parameter, *d*, in the range [0..1] that controls the weighting of expression change to connectivity used in the calculations. If *d *= 0, the ranking returned is based solely on the absolute value of the expression fold-change for that gene. For *d *= 1 we return the ranking based on connectivity. By setting *d *in the range [0..1] we interpolate between these two extremes. One advantage of our approach is that we apply the algorithm to the entire network, i.e. we do not require a pre-defined threshold of important genes. We simply require an experimentally determined expression change and some connectivity information for each gene, and based on this GeneRank will provide a re-ordering of genes in terms of their apparent importance. While "importance" may seem a rather vague concept in a biological context, we will show how it can be assessed objectively in the Testing the Algorithm section below. For full details of the algorithm and the random walk interpretation, the reader is referred to the Methods section.

### Data

In addition to the gene expression data, GeneRank uses a network or graph as input. We use the absolute value (the algorithm requires positive expression change values) of the gene expression data as a weight for each node in the graph and define the network connectivity by some other criterion. We use either Gene Ontology annotations or correlation coefficients, but there are many other possibilities, e.g., metabolic networks, transcription factor networks, or protein-protein interactions. We also used synthetic networks with controlled topological features for evaluation purposes. The three types of network were constructed as follows.

#### GO networks

Genes are connected if they share an annotation defined by the Gene Ontology. This defines three networks, one for each of the GO sections; Biological Process, Cellular Component and Molecular Function. We do not use the acyclic directed graph associated with the Gene Ontology, but assign leaf nodes as the annotations for each gene. A yeast diauxic shift experiment [12] was used to define the expression change vector. Data was chosen from the 20.5 hour time point when expression changes were largest. The mean degree, , and the clustering coefficient *C *[13], are given in Table 1 for each of the three networks. These are global graph properties which can be used to compare network topologies.

**Table 1:** Network Parameters

Network Parameters		
Network	*k*	C

Synthetic Network 1	40	0.0918
Synthetic Network 2	28	0.1034
Synthetic Network 3	40	0.0804
Biological Process	39	0.8636
Cellular Component	44	0.9461
Molecular Function	47	0.9444
Correlation Coefficient	155	0.5326

#### Correlation coefficient networks

A yeast stress data set consisting of 156 microarray experiments under a wide range of stress conditions was used to construct these networks. This data set is discussed in [14]. We randomly removed 15 experiments from the data set and each was used as the expression change vector. The correlation coefficient [15] was calculated for each gene pair using the reported expression changes for the remaining 141 experiments. Edges were defined in the network for pairs of genes with correlation *r *> 0.5. This data was taken from the Stanford Microarray Database [16]. Values for  and *C *are given in Table 1.

#### Synthetic networks

To allow control over the network structure, synthetic networks were defined with 1000 genes. The genes were split into two sets, *A *and *B*, where the genes in set *A *("changed genes") were allocated an expression change drawn from a *N*(2,1) distribution and the expression of the set *B *genes ("unchanged genes") were drawn from a *N*(0,1) distribution. Unless otherwise stated, |*A*| = 100 and |*B*| = 900 (the sizes of sets *A *and *B*). Edges were randomly assigned between genes with probabilities *p*_*A*_, *p*_*AB *_and *p*_*B*_, where these are the probabilities of two set *A *genes being connected, a set *A *gene being connected to a set *B *gene and two set *B *genes being connected, respectively. The clustering coefficient and the mean degree of the nodes depend on the values of *p*_*A*_, *p*_*AB *_and *p*_*B*_. Representative examples are listed in Table 1.

Synthetic network 1 is the standard case with equal expected degree across both sets and |*A*| = 100. Synthetic network 2 is the case where *E*_*deg*_*A *= 1.5*E*_*deg*_*B*. Also the relative connectivity () in both cases is 1 since this is where optimal results are observed (see relative expected degree section below). Synthetic network 3 is the case where |*A*| = 500 and again we have an equal expected degree across boths sets. Here the relative connectivity is 0.2618, again where optimal results are observed for this network (see Relative set size section below). The expected degree of a node in *A *is *E*_*deg*_(*A*) = (|*A*| - 1)*p*_*A *_+ |*B*|*p_AB _*and for set *B *is *E*_*deg*_(*B*) = |*A*|*p*_*AB *_+ (|*B*| - 1)*p*_*B*_.

**Figure 2:**
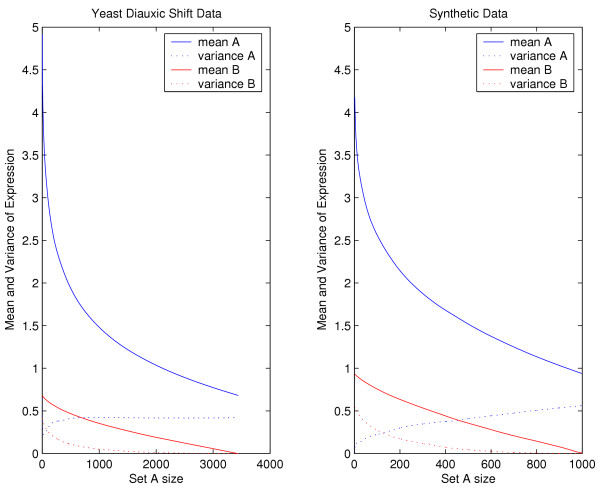
**Estimating *μ***. To justify drawing the expression of the set *A *genes from a *N*(2,1) distribution and the set *B *genes from a *N*(0,1) distribution, we compute the mean and variance for every possible size of set *A *and *B *in a synthetic network and compare this with an experimental data set.

To justify drawing the expression levels for sets *A *and *B *from *N*(2,1) and *N*(0,1) distributions, respectively, we compare the expression changes in a sample synthetic network to those in a yeast diauxic shift data set [12]. By measuring the mean and variance of the expression changes for every possible size of set *A *and *B *in both networks, we see that our method of assigning synthetic expression produces a distribution of values very similar to that observed in the diauxic shift experiment (see Figure 2).

### Testing the algorithm

#### Synthetic networks

Since we are trying to improve the ranking of genes produced in microarray experiment, we need to quantify the quality of the ranking produced by the algorithm. In the case of synthetic data, we know that all genes in set *A *should be ranked above the genes in set *B*, which gives us a basis for comparing the ranking from experimentally observed fold-changes with the re-ordered ranking produced by GeneRank. To quantify GeneRank results, we used the Area Under the Receiver Operating Characteristic Curve (AUC) [17,18]. This value describes how well the ranked list discriminates between genes in set *A *and set *B*. It will have a value of 0.5 if sets *A *and *B *are randomly mixed and a value of 1 if they are perfectly separated, i.e. if all the genes of set *A *("true changed genes") appear at the very top of the list. We emphasise that with *d *= 0 the algorithm is equivalent to ranking on pure fold-change.

The construction of synthetic networks allows us to obtain full control over the network structure. We experimented with various network parameters.

##### Relative connectivity and expression-connectivity weighting

We measure the relative connectivity as  since this is the expected number of connections in *A *divided by the expected number of connections between sets *A *and *B*. The expression-connectivity weighting parameter *d *is tested at 0.05 intervals in the range [0.05..0.95]. We vary both relative connectivity and expression-connectivity weighting in all tests along with one other variable. All results shown are averaged over 5 runs of the experiment and the AUC is calculated for each combination in the parameter space.

##### Relative expected degree

**Figure 3:**
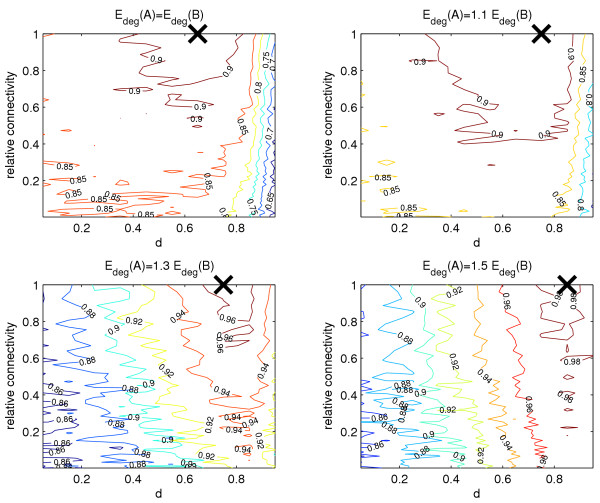
**Varying the relative expected degree between sets *A *and *B***. Here we are varying the expected degrees of sets *A *and *B*. The GeneRank algorithm provides slight improvement over pure expression based ranking when the expected degree of boths sets is equal. As the expected degree of set *A *becomes larger compared to that of set *B*, the improvement observed over expression ranking increases. We are measuring the AUC of the ranking (max = 1), averaged over 5 experiments. The lines on the diagrams indicate constant values of the AUC. The black cross indicates where the maximum AUC occurs, and hence shows for what values of *d *and relative connectivity the method works best.

We carried out a number of tests where *E*_*deg*_(*A*) = *αE*_*deg*_(*B*), for 1 ≤ *α *≤ 1.5. Here *E*_*deg*_(*A*) and *E*_*deg*_(*B*) denote the expected degree of a gene in sets *A *and *B*, respectively. Results are given in Figure 3. In all cases we see an improvement achieved by the algorithm compared to pure fold-change ranking. The effect is only slight when the expected degree of every node is equal throughout the network (*α *= 1), and increases for larger values of *α*. In addition, we never dramatically decrease the quality of the results for low values of *d *(*d *≤ 0.8). Also, the range of *d *for which we see an approximately constant high AUC increases as the relative connectivity increases.

A number of observations can be made from the experimental results where *E*_*deg*_(*A*) > *E*_*deg*_(*B*):

• The maximum AUC achieved by the algorithm increases as the difference between *E*_*deg*_(*A*) and *E*_*deg*_(*B*) increases. For the case where *α *= 1.5, we come particularly close to the maximum value of 1, (0.98).

• As the difference between *E*_*deg*_(*A*) and *E*_*deg*_(*B*) increases, we see less distortion of results even for unsuitably high values of *d*. This is consistent with the fact that increasing *d *gives connectivity a greater influence in the ranking.

• The maximum AUC achieved in each case occurs at larger values of *d *as the difference between the expected degrees increases. Again this is consistent with a high value of *d *corresponding to a greater weighting of connectivity on the algorithm.

• The improvement by the algorithm over expression change ranking is greater when the difference between the expected degree of both sets is greater.

To summarise these findings, a higher expected degree of set *A *genes compared with set *B *results in the algorithm producing a higher AUC, and hence more accurate results.

##### Relative set size

**Figure 4:**
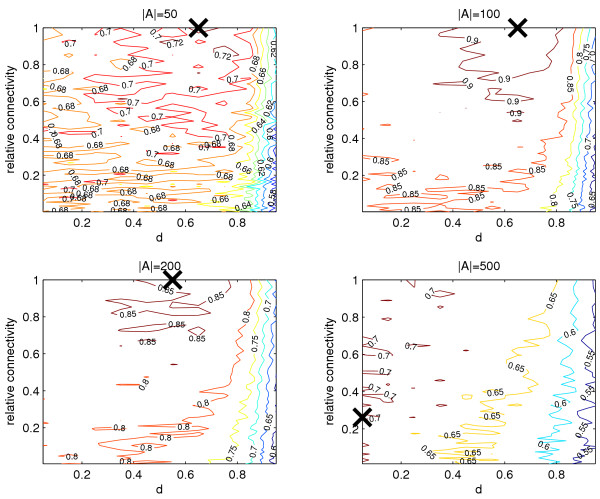
**Varying the relative sizes of sets *A *and *B***. Our previous tests used |*A*| = 100. Now |*A*| is varied and the AUC is again calculated, averaged over 5 experiments. The maximum AUC for each test is shown by the black cross. The expected degree of sets *A *and *B *are equal in all cases. The only case where the algorithm produces a deterioration of the expression based ranking is where *A *= 500, which is half of the total network size. In all other cases, the algorithm increases the AUC compared to that achieved by pure expression ranking. The highest AUC is achieved in the case where |*A*| = 100.

Four cases were investigated, where |*A*| = 50,100, 200 and 500. In each case |*B*| = 1000 - |*A*|, i.e. between 5 and 50% of the genes were defined as "differentially expressed". Networks were constructed to have equal expected degree across all nodes. Results are given in Figure 4. We see that the performance of the algorithm varies with |*A*|. The best results (highest AUC) are achieved at |*A*| = 100. In addition, an improvement over expression change ranking is observed for |*A*| = 50 and |*A*| = 200. As expected, with |*A*| = 500, where half of the total network is in set *A*, the algorithm performs most poorly and generally fails to give an improvement.

These results on synthetic networks suggest that for certain network structures GeneRank can achieve a significant improvement over ranking based on pure differential expression. The relative expected degree of sets *A *and *B *has a considerable effect on performance. In cases where the algorithm performs well (*E*_*deg*_(*A*) > *E*_*deg*_(*B*)), the optimal results occur when 0.75 ≤ *d *≤ 0.85. It is curious, but probably pure coincidence, that the value *d *= 0.85 is reportedly used by Google [1]. At such a high value of *d*, the algorithm is giving significant weight to connectivity information, as is appropriate considering the high level of experimental noise in the simulated expression data.

However, in our tests the quality of the results generally decreases for values of *d *beyond ≈ 0.85, showing that we do require some expression change information to make the best possible interpretation. It appears that although optimal results arise when there is some expression considered in the ranking, a major contributory factor to the success of the algorithm is the high relative degree of the genes that are differentially expressed. This structure is certainly not present in all biological networks. In the next section we explore whether suitable real networks can be identified.

#### GO networks

As described earlier, we construct the GO networks by defining an edge between two genes if they share an annotation allocated by the Gene Ontology Consortium. This allows us to construct three networks, one for each section defined by the Gene Ontology: Biological Process, Cellular Component and Molecular Function.

**Figure 5:**
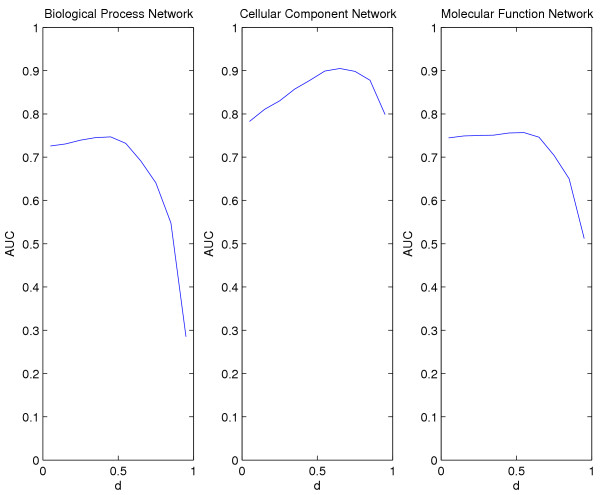
**Combining real connectivity with synthetic expression. **The network connectivity for each of the three GO networks: Biological Process, Cellular Component and Molecular Function were combined with synthetic expression data. In each case, the 300 most down-regulated genes which were defined using the real yeast diauxic shift data were allocated expression from a *N*(2,1) distribution and the remainder of the genes were given expression from a *N*(0,1) distribution. Again we measure the AUC for the ranking. In all cases an improvement over expression ranking is observed for lower values of *d *although for the Molecular Function network this change is slight. Applying the algorithm to the Cellular Component network achieves the highest overall AUC. The results begin to decrease in quality for *d *> 0.55, except for the Cellular Component network where the results decrease for *d *≈ 0.65.

An initial test combined the real network connectivity with synthetic expression changes. We ordered the genes based on expression change in the yeast diauxic shift experiment and allocated the top 300 down-regulated genes to set *A*. We know from the synthetic network testing that it is preferable for the algorithm to have a higher expected degree of set *A *compared to set *B*. This is the case with each of the three networks where the set *A *genes were chosen in this way. Synthetic expression was assigned as before to the genes in sets *A *and *B*. This allowed us to quantify the results produced by the algorithm. We used the algorithm to find the AUC for 0.05 ≤ *d *≤ 0.95. Results for the three networks are shown in Figure 5, and in each case we see that the algorithm is able to improve on expression change ranking. In particular, with values of 0.05 ≤ *d *≤ 0.5 we increase the AUC for all cases, and for general use of the algorithm we would therefore suggest *d *= 0.5 would be an appropriate choice for *d*. This increase of AUC is only slight in the case of the Molecular Function network, but is more dramatic for the Cellular Component network. Also, on average, the AUC is high for the Cellular Component network, and is higher for all values of *d *than is obtained for the other networks.

**Figure 6:**
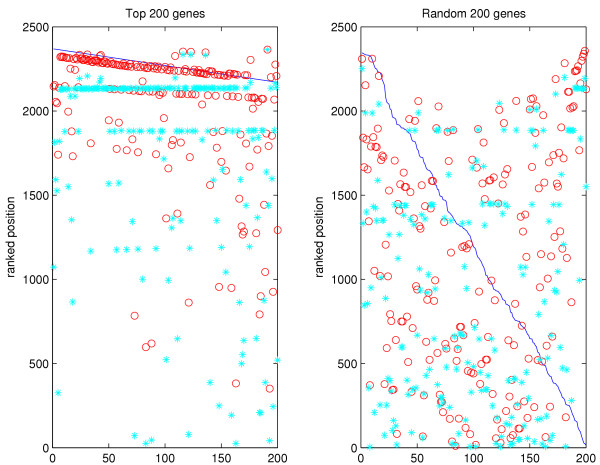
**GO networks: testing the 'boosting' ability of the algorithm**. An experiment was carried out to assess how well the algorithm is able to increase the relative ranking of a gene based on its connections to other highly changed genes. The top 200 most changed genes are set in turn to have a differential expression of 0. If the ranking were based on pure differential expression only, each gene would appear at the bottom of the list. By PageRanking, we raise the position of the gene closer to its original ranking. The same effect is not observed when a random 200 genes are chosen. The majority of these genes will not have connections to other highly changed genes. The blue line represents the original expression ranked position, the red circles show the original GeneRanked position and the blue asterisks show the modified GeneRanked position.

To check if GeneRank produces a gene ranking which is robust to noise we conducted a further experiment using the GO networks. Real experimental data were used throughout. The Cellular Component network was used in this experiment. For each of the top 200 genes sorted by differential expression, we set its expression change to 0 in turn (i.e., defined it as "unchanged") and determined if the GeneRank algorithm was able to pick up this anomaly and consequently move the gene towards its original place in the ranked list. The premise here is that its connections to other highly changed genes will boost the artificially altered gene in the ranking. The same experiment was done for 200 randomly selected genes. The results are given in Figure 6.

To quantify the results we calculated the quality index *B *as



where alt_PR is the GeneRanked position after the expression of the gene has been artificially altered, orig_exp_rank is the original expression-based position in the list, and alt_exp_rank is the expression-based position after the differential expression has been set to 0.

In the case where we are altering a gene in the top 200, a 'boosting' effect is observed and the ranked position after the fold-change has been moved towards the original ranked position. We can observe groups of genes which are boosted to the same level (shown by 'lines' of blue asterisks). It is likely that these genes are a completely connected subgraph, which results in all genes being given the same ranking. Altering genes which were originally ranked in the top 200, we achieve *B *= 0.7728. This effect is not observed for a random set of genes (*B *= 0.4165).

#### Correlation coefficient networks

**Figure 7:**
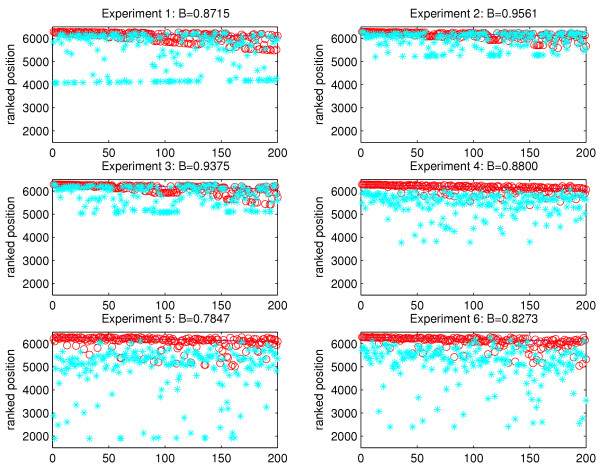
**Correlation Coefficient networks: testing the 'boosting' ability of the algorithm. **The same experiment as in Figure 6 was carried out on the correlation coefficient networks. In each case the network connectivity is identical but the expression change vector, used as input to the algorithm, is randomly chosen to be one experiment from the stress data set. The x-axis represents the top 200 genes when ranked using expression change information. We calculate a 'boosting' measure to quantify how much we increase the relative rank of each gene after it has been altered. In this case, each gene was changed to have expression change 1. The large values for *B *(max = 1) indicate that the algorithm achieves a high level of 'boosting' due to the connections that the altered genes have to highly changed genes. The blue line represents the original expression ranked position, the red circles show the original GeneRanked position and the blue asterisks show the modified GeneRanked position.

Using the correlation coefficient network defined by the stress data set [14] we carried out the same experiment to check the robustness of the ranking produced by the algorithm. Here we have one network with different expression change vectors to be used as input to the algorithm. Each of the 15 experiments in the data set, which were not used in the network construction, was used as the input expression vector in turn. Results for six representative experiments are shown in Figure 7. The expression change of each gene was set to 1 in turn, i.e. defining it as "only slightly changed".

Again we calculate a value of our quality index *B *as described above. As a result of the high degree of the top 200 genes, we see that a high level of 'boosting' is achieved, as demonstrated by the high values of *B*. In other words, we are able to significantly raise the position of the altered gene in comparison with the ranked position that would have been observed had the standard expression change ranking been used.

## Conclusion

The purpose of this work was to explore the possibility of adapting the PageRank algorithm, used by Google in assessing the importance of web pages, for the task of prioritizing the 'importance' of genes in a microarray experiment. Our new algorithm, GeneRank, allows connectivity and expression data to be combined to produce a more robust and informed summary of an experiment, compared to the standard procedure of basing the importance of a gene on its measured expression change. Although we use expression change values as expression data, this is not restricted, and some other means of capturing the expression information may also be used. GeneRank can be justified theoretically and has been tested on synthetic data, experimental data and a combination of both. The algorithm has a single parameter, *d*, that controls the relative weighting of expression and connectivity information. A value *d *= 0 ranks genes based on pure expression information and a value *d *= 1 ranks on pure connectivity degree. The optimal value of *d *is data-dependent, but based on our results we suggest *d *= 0.5 for general use. With *d *= 0.5 we observed no deterioration and generally an improvement over ranking based on pure expression change in the case where we combined real connectivity information with synthetic expression changes. GeneRank is simple to implement, gives a principled approach to combining different data types, and is a novel instance of applying search engine technology to this important task. We note that GeneRank results are not designed to replace the actual expression measurements, but should be used alongside the results with additional biological knowledge, to draw attention to unusual structures within the data. For example, a gene which is not viewed as important from the microarray results alone but is highly ranked in the GeneRank results, should be given further biological consideration.

While the improvement of gene rankings upon application of GeneRank is already significant in the examples presented, it may become even more so once comprehensive high-quality biological network information becomes available. Of particular interest in that respect will be transcriptional regulatory networks, such as are now being generated by technologies like ChIP-chip (see [19-21] for early examples using yeast as a model organism). As discussed above, the information encoded in such regulatory networks will be intuitively amenable to GeneRank analysis. It will also re-introduce an element of directedness into the network, moving it even closer to the original PageRank application.

## Methods

### The original algorithm

We summarize the basic PageRank algorithm which was developed by Larry Page and Sergey Brin at Stanford University [2] and forms the basis of the successful search engine Google. Further details may also be found in [1,22].

PageRank assigns a measure of relevance or importance to each web page, allowing Google to return high-quality pages in response to a user query. The algorithm is designed to be robust to methods of deception, where web page designers attempt to artificially boost the PageRank of their page by altering the local link structure. Robustness follows from the recursive nature of the algorithm, where a page is highly ranked if it is linked to by other highly ranked pages. A link from page *i *to page *j *is regarded as a "vote of confidence" for page *j *from page *i*. The algorithm views the web as a directed graph *G*(*V, E*), where the *N *nodes *V *are the web pages and the edges *E *represent the links between pages. This information can be stored in an adjacency matrix, *W *∈ *R*^*N *× *N*^, where *w*_*ij *_= 1 if there is a link from page *i *to page *j *and *w*_*ij *_= 0 otherwise. We define deg_*i *_:=  to be the *degree *(more precisely, the out-degree) of the *i*th page. Suppose we have assigned an initial ranking **r**^[0] ^∈ ^*N*^. The PageRank algorithm proceeds iteratively, updating the ranking for the *j*th page from  to  according to the formula



Here  denotes the ranking of page *j *at the *n*th iteration and *d *∈ (0,1) is a fixed parameter. The value *d *= 0.85 appears to be used by Google [1,2]. We see from (1) that the rank of a page depends on the rank of all pages that link to it. Scaling by 1/deg_*i *_in the summation ensures that each page has equal influence in the voting procedure. Each page gets a rank of 1 - *d *automatically and also gets *d *times the votes given by other pages. Iterating to convergence in (1) is equivalent to solving for **r **∈ ^*N *^in the linear system

(*I *- *dW*^*T *^*D*^-1^)**r **= (1 - *d*)**e**,     (2)

where *I *is the identity matrix, *W*^*T *^is the transpose of *W*, *D *= diag(deg_*i*_) and **e **∈ ^*N *^has all *e*_*i *_= 1. Applying PageRank is equivalent to applying the Jacobi iteration [23] to (2), and convergence to a unique solution **r **is guaranteed under the condition

*ρ*(*dW*^*T*^*D*^-1^) < 1,     (3)

where *ρ*(·) denotes the spectral radius. The convergence condition (3) holds for any 0 <*d *< 1.

### A random walk interpretation

The PageRanking process has an alternative interpretation in terms of a random walk [1,2,22]. Suppose that a random walker is currently at page *i*. On the next step the walker

**teleports: **with probability 1 - *d *moves to a new page, chosen uniformly over all web pages, or,

**surfs: **with probability *d *moves to a page that is linked to from page *i*; in this case each page *j *such that *w*_*ij *_= 1 is equally likely to be chosen as the destination.

The PageRank vector **r**, when normalised so that its components sum to one, corresponds to the invariant measure for this process. In other words, *r*_*j *_is the long-time proportion of visits made to page *j*. A further interpretation based on mean hitting times rather than invariant measures is given in [24]. The biological implication of the random walk interpretation is discussed in the description of the algorithm in the Results and Discussion section.

### The modified algorithm: GeneRank

The PageRank idea translates intuitively to the analogous situation of gene expression analysis. Instead of producing a ranked list of web pages, we produce a ranked list of genes. PageRank views hyperlinks as votes of confidence, so we similarly allow functional connections to boost rank. Just as PageRank counts votes from a highly-ranked page as more influential than votes from a lowly-ranked page, we will allow connections to genes with high differential expression to carry greater significance than connections to genes with low differential expression. Figure 1 gives a graphical view of the concept.

PageRank gives each web page a rank of (1 - *d*) "for free". We will adapt this to give each gene a rank of (1 - *d*)ex_*i*_, where ex_*i *_is the absolute value of the expression change for gene *i*. Letting  denote the ranking of gene *j *after the *n*th iteration, we take initial ranking **r**^[0] ^= **ex**/||**ex**||_1_, where ||·||1 denotes the vector 1-norm. Then we let



Here *W *∈ ^*N *× *N *^is the connectivity matrix for the gene network, so *w*_*ij *_= *w*_*ji *_= 1 if genes *i *and *j *are connected and *w*_*ij *_= *w*_*ji *_= 0 otherwise.

We remark that this iteration may also be motivated from the viewpoint of *personalised PageRanking *[1,2], where teleporting jumps in the random walk process are biased towards a user's preferred locations – here, we are biasing according to expression level.

The iteration (4) corresponds to Jacobi on the system

(*I *- *dW*^*T *^*D*^-1^)**r **= (1 - *d*)**ex**,     (5)

and, because the iteration matrix has not been altered, the condition that convergence is guaranteed for all 0 <*d *< 1 continues to hold. Since *W *is symmetric as the network is undirected, we could replace *W*^*T *^by *W*. This is unlike the original algorithm, where a directed network is used.

In summary, the GeneRank algorithm is finding the customised ranking vector **r **defined by the linear system (5). A Matlab implementation of the algorithm is available in the additional file geneRank.m The random walk interpretation carries through to this more general setting. If the teleporting step is re-defined so that the destination gene is not chosen uniformly over the whole set, but rather is chosen with probability proportional to absolute expression level, then **r **in (5), suitably scaled, is the invariant measure. Overall, we have a true generalization of PageRank in the sense that (a) the algorithm has both "vote of confidence" and "random walk" interpretations and (b) for the case where all ex_*i *_= 1 we recover the original PageRank algorithm.

It is trivial to check that with the choice *d *= 0 the system (5) has solution **r **= **ex**. In this case the genes are ranked purely on expression level. We will now study the other extreme, where *d *= 1, and show that this case may be regarded as ranking purely on connectivity.

For *d *= 1, the iteration (4) becomes



and the system (5) for the corresponding fixed point becomes

(*I *- *W^T ^D*^-1^)**r **= **0**.     (7)

First, we show that the sum of the rankings is preserved by the iteration. From (6),



Also, it is clear from (6) that the iteration preserves the nonnegativity of the initial ranks; that is,  ≥ 0. Next, we note that **deg**/||**deg**||_1 _is a fixed point of (6). To see this, put **r**^[*n*-1] ^= **deg**/||**deg**||_1 _in the right-hand side of (6) to obtain



Now, we observe that *ρ*(*W*^*T*^*D*^-1^) ≤ ||*W*^*T*^*D*^-1^||_1 _= 1, and hence all eigenvalues of *W*^*T*^*D*^-1 ^are less than or equal to 1 in modulus. Because *W *is symmetric, we have *W*^*T*^*D*^-1^**deg **= *W*^*T*^**e **= **deg**, showing that there is at least one eigenvector, **deg**, corresponding to eigenvalue 1. Suppose now that λ = 1 is a simple eigenvalue of *W*^*T*^*D*^-1 ^and that r* with ||r*||_1 _= 1 is another solution of (7). Then



So r* - **deg**/||**deg**||_1 _is an eigenvector of *W*^*T*^*D*^-1 ^corresponding to eigenvalue 1. It follows that r* - **deg**/||**deg**||_1 _must be a multiple of **deg **and hence r* = ± **deg**/||**deg**||_1 _. We may summarize our findings in the following result.

**Result **If the eigenvalue λ = 1 of *W*^*T*^*D*^-1 ^is simple, then **r **= **deg**/||**deg**||_1 _is the unique solution of (7) that satisfies the required constraints ||**r**||_1 _= 1 and *r*_*i *_≥ 0.

Overall, we conclude that the extremal parameter values *d *= 0 and *d *= 1 represent ranking by pure expression level and pure degree, respectively, and hence by changing the value of *d *we may interpolate between these two extremes.

## Abbreviations

ROC – Receiver Operating Characteristic

AUC – Area Under ROC Curve

GO – Gene Ontology

## Authors' contributions

JLM implemented the algorithm, performed the experiments, and drafted the manuscript. RB provided biological datasets and interpretation, and participated in the experimental design. DRG contributed to study coordination and continuous discussions. DJH first conceived of the application of PageRanking to biological data, and participated in study design and supervision. All authors read and approved the final manuscript.

## Supplementary Material

Additional File 1 **The Matlab GeneRank implementation**. The file contains a Matlab implementation of the GeneRank algorithm. The file requires a matrix describing the network connectivity and a vector of expression changes for each gene. The output is the vector of rankings for each gene. Click here for file

Additional File 2 **A Matlab .mat file containing sample GO networks and expression change vectors**. This file can be loaded into Matlab using the command load G0_matrix. This will load three matrices (w_All, w_Up and w_Down) and three expression change vectors (expr_data, expr_dataUp and expr_dataDown) into the current workspace. These matrices were constructed using the all three sections of the Gene Ontology, where a link is present between two genes if they share a GO annotation. Only genes which are up-regulated are included in w_Up and only down-regulated in w_Down. The GeneRank algorithm should be used with the corresponding matrix and expression change vector, e.g. the command ranking = geneRank(w_Up, expr_dataUp,d) would be used to calculate the ranking of the up-regulated genes in the experiment. Click here for file
